# Generation of dendritic cell-based vaccines for cancer therapy

**DOI:** 10.1038/sj.bjc.6600316

**Published:** 2002-05-03

**Authors:** G Reinhard, A Märten, S M Kiske, F Feil, T Bieber, I G H Schmidt-Wolf

**Affiliations:** Klinik und Poliklinik für Dermatologie der Rheinischen Friedrich-Wilhelms-Universität Bonn, Germany; Medizinische Klinik und Poliklinik I der Rheinischen Friedrich-Wilhelms-Universität Bonn, Germany

**Keywords:** dendritic cell, melanoma, vaccine, peptide

## Abstract

Dendritic cells play a major role in the generation of immunity against tumour cells. They can be grown under various culture conditions, which influence the phenotypical and functional properties of dendritic cells and thereby the consecutive immune response mainly executed by T cells. Here we discuss various conditions, which are important during generation and administration of dendritic cells to elicit a tumouricidal T cell-based immune response.

*British Journal of Cancer* (2002) **86**, 1529–1533. DOI: 10.1038/sj/bjc/6600316 www.bjcancer.com

© 2002 Cancer Research UK

## 

Cytotoxic T-cells (CTL) are the most efficient cells concerning defence against tumour cells (Lanzavecchia, 1993). One of the major questions in tumour immunology is, how an efficient CTL-response can be generated to elicit an antigen-specific and protective T-cell response *in vivo*. It has been shown that the immune response to tumour antigens and other antigens is altered in patients with cancer. These alterations concern many elements of the immune response and prevent effective proliferation of tumour-antigen specific T cells and their subsequent recognition of tumour cells. In recent studies antigen-presenting cells (APC) have been shown to play a crucial role in the induction of tumour-protective immune responses by generating tumour-specific T cells (Lanzavecchia, 1993). Antigen receptors (TCR) of tumour-specific T cells recognise tumour-associated peptides that are presented in the context of HLA class-I or class-II molecules by the APC. Successful recognition of tumour–antigen by the T-cell is not only dependent on TCR-peptide-HLA-interaction, but other co-stimulatory signals must be provided to prevent anergy (Schwartz, 1990). These are mainly CD80/CD86–CD28- or CD40–CD40L-interactions (Bennett *et al*, 1998). These interactions do not only underline the importance of T cells, but also the significant role of dendritic cells (DC), which are the most potent antigen-presenting cells among others like monocytes, macrophages and B cells.

Several *in vitro* and *in vivo* studies showed the ability of vaccination with DC to elicit tumour-specific T-cell immunity (Schuler and Steinman, 1997). This result implies that (1) DC might be just another altered element of the immune system or (2) DC are able to overcome tumour-protective alterations in cancer patients by inducing effective CTL response or (3) both. In this context a phenotypic and functional dichotomy of DC in DC1 and DC2 appears to be of importance. DC1 and DC2 cells were found to produce different cytokines and thereby induce T_H_1 and T_H_2 differentiation, respectively. The lymphoid-related DC (DC2) are CD11c^−^ and have been shown to induce a tolerating response *vs* tumour cells by activating mainly T_H_2 cells, whereas myeloid-derived DC (DC1) are immunostimulatory via T_H_1 cells. Development of T_H_2-promoting DC2 cells is inhibited by cytokines (IL-4) produced by T_H_2 cells. In contrast, development of T_H_1-promoting DC1 cells is enhanced by the T_H_2 cytokine IL-4 (Banchereau and Steinman, 1998; Rissoan *et al*, 1999).

Vaccination with peptide- or lysate-pulsed DC showed the clinical efficiency in the induction of a curative tumour-specific therapy in metastatic melanoma and other malignancies (Table 1

**Table 1:**
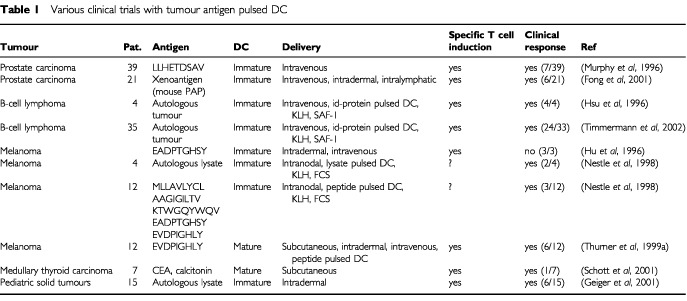
Various clinical trials with tumour antigen pulsed DC

; Thurner *et al*, 1999a). In a study performed with peptide- and lysate-pulsed DC five out of 16 patients showed at least a partial remission, two of them a complete remission (Nestle *et al*, 1998). Monocytes drawn from peripheral blood were grown in the presence of a cytokine cocktail (GM–CSF, IL-4, IL-1β, IL-6, TNF-α, PgE_2_) to DC, which display mainly the DC1-phenotype. Subsequently, DC were pulsed with peptides or autologous tumour-lysate and injected back into the patient with different clinical outcome.

Several factors seem to influence a successful vaccination by peptide- or lysate-pulsed DC: (1) Generation of DC; (2) Selection of tumour-antigen pulsing of DC; (3) Transfection into DC; and (4) Route of application of DC.

### Generation of DC

Physiologically, human DC are mainly localised in tissue and represent only a small portion of less than 0.5% of peripheral blood leukocytes. For therapeutical purposes large numbers of DC are needed.

DC can either be generated from proliferating CD34^+^ bone marrow precursor cells (Caux *et al*, 1996) – which differentiate under a variety of different cytokines including SCF, Flt3, GM–CSF, TGF-β and TNF-α – or from non-proliferating peripheral CD14^+^ cells (monocytes) (Sallusto and Lanzavecchia, 1994). Usually, CD34^+^ precursors mobilised by G-CSF are isolated by leukapheresis to obtain high numbers of peripheral cells for therapeutical purposes. These cells seem to be more efficient in the activation of tumour-specific CTLs than CD14^+^ derived DC (Mortarini *et al*, 1997). CD34^+^ cells expand 10–30-fold. Yields of 5×10^6^ cells per leukapheresis are typically obtained. In contrast, monocytes are abundantly present in peripheral blood and can be easily obtained by peripheral blood drawings or leukapheresis. Protocols for the generation of large amounts of monocyte-derived DC are known since 1994 (Romani *et al*, 1994; Sallusto and Lanzavecchia, 1994) and have been used for both experimental and therapeutical purposes. Here, leukocytes are prepared from peripheral blood using Ficoll–Hypaque density centrifugation. Monocytes are isolated by an adherence step and subsequently cultured in the presence of GM–CSF, IL-4 and 10% FCS or alternatively – under serum free conditions (Jonuleit *et al*, 1997; Thurner *et al*, 1999b) – with 1% autologous plasma for 7 days (Figure 1

**Figure 1:**
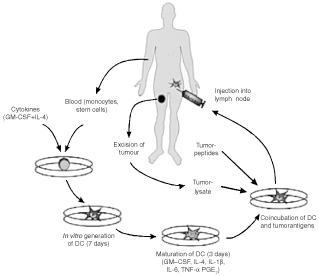
Schematic diagram of dendritic cell generation.

). After 1 week the yield of DC generated varies from about 25 to 50% of the starting population. Yields of 0.5–2.0×10^6^ cells per 10 ml blood are typically obtained. Adherent cells show cytoplasmic processes typical for DC. After co-culturing with immunologic effector cells DC form typical cluster.

DC display several antigens on their cell surface, all of which are characteristic, but not specific. The most typical markers at present are HLA-class-I-, -class-II-molecules and co-stimulatory markers (CD80, CD86). Immature DC – obtained after 7 days of culture with GM–CSF and IL-4 – can be grown to mature DC by co-culturing with TNF-α, IL-6, IL-1β, and PGE2 or, alternatively, with a so-called monocyte conditioned medium (MCM) for another 3 days (maturation phase) (Thurner *et al*, 1999b). In contrast to immature DC, mature DC are much more potent in inducing T_H_1 and CTL responses *in vitro* and are resistant to immunosuppressive effects of tumour-derived IL-10 (Steinbrink *et al*, 1999). Therefore, mature DC have been used in recent vaccination protocols (Thurner *et al*, 1999a). Table 2

**Table 2:**
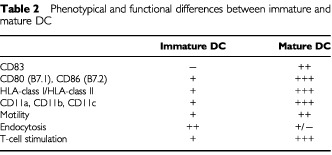
Phenotypical and functional differences between immature and mature DC

shows major phenotypical and functional differences between immature and mature DC.

### Selection of tumour-antigen pulsing of DC

Small antigenic peptides (consisting of 8–10 amino acids) are loaded directly from outside the cell on HLA-class-I-molecules, whereas tumour-lysates – as a protein or oligopeptide (>10 amino-acids) – are internalised by endocytosis into the antigen presenting cell, processed and then presented with the HLA class-II-molecule. Cross-presentation of tumour-lysate is possible, i.e. the presentation of proteins or oligopeptides with HLA class-I-molecules (Bennett *et al*, 1997). This implies that pulsing with tumour-lysate or oligopeptides is also able to activate CD8^+^ T-cells immediately by the HLA-class-I-pathway and not only CD4^+^ T-cells by the ‘conventional’ HLA-class-II-pathway. CD40/CD40L signalling via DC/CD4^+^ T-cell-interaction is able to pre-activate DC temporarily. Subsequently these CD4^+^ T-cell-pre-activated DC can generate cytotoxic responses in CD8^+^ T cells (Bennett *et al*, 1998).

The selection of the peptide used for vaccination, is influenced by several factors: type of tumour, HLA class-I or -II of the patient, successful induction of CTL-response *in vitro* or *in vivo*, etc. Today, more than 50 melanoma-associated epitopes are known, which can be recognised by T cells. These epitopes are presented via different HLA class-I- and HLA class-II-molecules (e.g. HLA A1, HLA A2, HLA DR4, …). An optimal selection of these epitopes allows the treatment of almost 100% of patients with peptide-pulsed DC for the case of malignant melanoma.

Vaccination of tumour–patients with a single peptide can result in peptide-specific cytotoxicity. In these cases tumour escape mechanisms may be a problem, for example by the loss of tumour-associated epitopes or of essential antigen presenting molecules. This problem can be circumvented by the usage of polyvalent vaccines in a single patient, i.e. the application of tumour lysates or the application of several peptides. This phenomenon can also be observed in a subgroup of a trial conducted by Nestle *et al* (1998). Patients treated with tumour–lysate showed highest response rates (50%, two out of four) compared to patients treated with peptides alone (25%, three out of 12) (Table 1).

Tumour-associated peptides do bind with a defined affinity both to the HLA molecules (i.e. HLA class-I and HLA class-II) and the TCR. Whether this peptide is useful for therapeutical purposes or not mainly depends on the degree of its affinity. Low affinity to the HLA molecules is synonymous with low potency in CTL induction, whereas high affinity means high potency in CTL induction (Sette *et al*, 1994). Therefore, vaccination was performed with a so-called heteroclitic peptide. Original melanoma-associated peptides show a substitution of one amino acid with another at the same position. Heteroclitic peptides, that are changed at the HLA-binding motif to achieve a higher affinity between the HLA and the peptide, are potent immunogens. They are able to elicit cross-reactivity with the original peptide, because the TCR-binding motif remains unchanged. As a consequence a tumour-protective immune response against the original peptide can occur after vaccination with the heteroclitic peptide (Rosenberg *et al*, 1998).

### Transfection into dendritic cells

Enhancing the immunogenicity of tumour cells is an interesting approach to cancer gene therapy (Schmidt-Wolf *et al*, 1994). Cytokine genes have been used in most instances to enhance tumour immunogenicity (Schmidt-Wolf and Schmidt-Wolf, 1996). DC are attractive targets of gene transfer since DC are easily accessable and since these cells seem to be sensitive to immunologic strategies. For further enhancement of the antigeneic presentation by DC various genes like the genes for interleukin-7 (Westermann *et al*, 1998), GM–CSF, interleukin-12, interferon-gamma and interferon-alpha (Tüting *et al*, 1998) have been transfected into DC. Up to 10% transfection efficiencies using electroporation for gene transfer into CD83^+^ mononuclear cell derived DC were reported. Other non-viral techniques produce robust DC transfection with 17% of monocyte-derived DC using cationic peptide or report the ability of using lipofection in principle.

Higher efficiencies can be achieved using viral vectors. Adenoviral vectors seem to be the most efficient transfection method (Mulders *et al*, 1998). Fifty to 85% transfected CD83^+^ DC generated from PBMC were reported. Thirty to 40% of precursor DC derived from human umbilical cord blood can be transduced using adenoviral vectors without cytopathic effect. With the aid of liposome-mediated infection, gene transfer into CD83^+^ DC resulted in more than 90% of the cells transduced. Using a protocol with UV-irradiated adenoviruses similar results can be obtained without addition of liposomes (Mulders *et al*, 1998; Märten *et al*, 2001). Adenoviral vectors can also be used for transduction of CD34^+^ cell derived DC (Bregni *et al*, 1998). For retroviral vectors a transduction efficiency of 10–30% has been reported; other groups described a resistance of DC to transduction by retroviral vectors. Recently, there were reports of using other viral vectors like fowlpox virus, lentivirus, avipoxvirus or vaccinia virus (Di Nicola *et al*, 1998).

### Route of application of DC

Cell-based immunotherapy strategies using peptide- or lysate-pulsed DC require interaction between DC and T cells. Physiologically, bone marrow-derived DC or their progenitors migrate to tissues of inflammation, internalise antigens and subsequently reach the paracortex of the lymph nodes (Steinman, 1991) and the periarteriolar lymphoid sheath of the spleen (PALS). Here, DC prime naive T cells. The optimal route of administration of *in vitro*-cultured DC for migration to T-cell-rich sites is unknown, particularly since migratory capacity of cultured tumour-antigen-pulsed DC may be altered. Possible routes of administration are *intradermal, subcutaneous, intranodal, intravenous* and *intraperitoneal injection* of DC. Except intraperitoneal injection, which was performed in animal studies only, all of these applications have been employed in human cell-based vaccination protocols (Table 1). To examine migration patterns of DC, they were radioactively labelled with indium-111. Subsequently, tumour antigen-pulsed DC were administered by an intravenous, subcutaneous, or intradermal injection in patients with metastatic malignancies (Morse *et al*, 1999): Three patients received intravenous injection, four patients received intradermal or subcutaneous injections simultaneously on both sides of their body. *Intravenous* injection revealed highest activity in the lungs after 1 min, which decreased continuously and redistributed after 24 h to highest spleen and liver activities. No activity was found in lymph nodes and tumour. *Subcutaneous* injection showed no activity in the lymph nodes. It could not be detected where the injected DC remained. *Intradermal* injection revealed highest activity in the draining lymph nodes after 24 h. Only 0.1–0.4% of relative activity was found here, i.e. only 4000 of 10^6^ injected cells reached the lymph node in contrast to 10^6^ of 10^6^ cells after successful intranodal injection. In conclusion, greatest activity in lymph nodes was only found after intradermal injection. Subcutaneous injection seemed to be ineffective and intravenous injection showed accumulation in the spleen as a T-cell-rich area. Technetium-labelled immature monocyte-derived DC have also been shown to migrate rapidly to the draining lymph nodes after intradermal injection (Thomas *et al*, 1999). Nonetheless, injecting DC directly into the lymph node seemed to deliver highest numbers of DC in T-cell-rich area, although this mode of application may destroy the normal architecture of the lymph node.

Studies where antigens were continuously injected into a lymph node, for example by a pump, have yet to be performed in humans. It has been shown that route and kinetic of peptide administration determine its immunogenicity. This may also be the case for DC administration.

### Adverse effects

Peptide- or tumour-lysate pulsed DC are able to induce CTL-response in patients with malignant melanoma. Peptide or tumour-lysate antigens, used for DC vaccination, are normally not restricted to tumour tissue, but can be found at least partially on healthy tissue. Therefore a risk for the development of autoimmune diseases exists, that has been shown in animal models (Ludewig *et al*, 2000). Pilot clinical studies in humans could not find clinical signs of auto-immunity except vitiligo and the occurrence of auto-antibodies (anti-TSH-receptor-Ab, ANA). Unexpectedly, vaccination with tumour-lysate pulsed DC did not show a higher incidence of auto-immunity than vaccination with peptide-pulsed DC. The occurrence of IgG, IgM and IgE antibodies to bovine serum albumin (BSA) causing anaphylaxis after vaccination with human peptide-pulsed DC was reported (Mackensen *et al*, 2000). Therefore, for therapeutical *ex vivo* applications the use of serum-free generated DC was recommended.

More frequently systemic flu-like symptoms occurred: fever or painful swelling of the injected lymph node (after intranodal injection). After intradermal injection of the pulsed DC swelling, itching and erythema at the injection site could be detect. These reactions regressed within 48–72 h.

Induction of tolerance against tumour cells may be a problem, although recent studies pointed out the possible therapeutical value of DC vaccination.

### Future developments

In conclusion, DC are able to increase the tumouricidal activity of immunologic effector T cells against tumour cells. This ability depends on several factors as has been discussed. Nonetheless, factors that influence effectively DC-activation of T cells against tumour cells have to be optimised. The generation of DC-subtypes that are more effective than those known today seems to be promising in inducing tumouricidal, specific immune responses not only in patients with malignant melanoma, but other malignant diseases. DC-based cell therapy will not only be conducted in patients with distant metastases, but also in patients with minimal residual disease or in adjuvant settings for high risk situations, where tumour-load is low and therefore tumours can be recognised specifically and eliminated effectively by DC-activated immunologic effector cells.

## References

[bib1] BanchereauJSteinmanRM1998 Dendritic cells and the control of immunity Nature 392 245252 9521319 10.1038/32588

[bib2] BennettSRCarboneFRKaramalisFFlavellRAMillerJFHeathWR1998 Help for cytotoxic-T-cell responses is mediated by CD40 signalling Nature 393 478480 9624004 10.1038/30996

[bib3] BennettSRCarboneFRKaramalisFMillerJFHeathWR1997 Induction of a CD8+ cytotoxic T lymphocyte response by cross-priming requires cognate CD4+ T cell help J Exp Med 186 6570 9206998 10.1084/jem.186.1.65 PMC2198964

[bib4] BregniMShammahSMalaffoFDi NicolaMMilanesiMMagniMMatteucciPRavagnaniFJordanCTSienaSGianniAM1998 Adenovirus vectors for gene transduction into mobilized blood CD34+ cells Gene Ther 5 465472 9614570 10.1038/sj.gt.3300620

[bib5] CauxCVanbervlietBMassacrierCDezutter-DambuyantCde Saint-VisBJacquetCYonedaKImamuraSSchmittDBanchereauJ1996 CD34+ hematopoietic progenitors from human cord blood differentiate along two independent dendritic cell pathways in response to GM-CSF+TNF alpha J Exp Med 184 695706 8760823 10.1084/jem.184.2.695 PMC2192705

[bib6] Di NicolaMSienaSBregniMLongoniPMagniMMilanesiMMatteucciPMortariniRAnichiniAParmianiGDrexlerIErfleVSutterGGianniAM1998 Gene transfer into human dendritic antigen-presenting cells by vaccinia virus and adenovirus vectors Cancer Gene Ther 5 350356 9917089

[bib7] FongLBrockstedtDBenikeCBreenJKStrangGRueggCLEnglemanEG2001 Dendritic cell-based xenoantigen vaccination for prostate cancer immunotherapy J Immunol 167 71507156 11739538 10.4049/jimmunol.167.12.7150

[bib8] GeigerJDHutchinsonRJHohenkirkLFMcKennaEAYanikGALevineJEChangAEBraunTMMuleJJ2001 Vaccination of pediatric solid tumor patients with tumor lysate-pulsed dendritic cells can expand specific T cells and mediate tumor regression Cancer Res 61 85138519 11731436

[bib9] HsuFJBenikeCFagnoniFLilesTMCzerwinskiDTaidiBEnglemanEGLevyR1996 Vaccination of patients with B-cell lymphoma using autologous antigen-pulsed dendritic cells Nat Med 2 5258 8564842 10.1038/nm0196-52

[bib10] HuXChakrabortyNGSpornJRKurtzmanSHErginMTMukherjiB1996 Enhancement of cytolytic T lymphocyte precursor frequency in melanoma patients following immunization with the MAGE-1 peptide loaded antigen presenting cell-based vaccine Cancer Res 56 24792483 8653680

[bib11] JonuleitHKuhnUMullerGSteinbrinkKParagnikLSchmittEKnopJEnkAH1997 Pro-inflammatory cytokines and prostaglandins induce maturation of potent immunostimulatory dendritic cells under fetal calf serum-free conditions Eur J Immunol 27 31353142 9464798 10.1002/eji.1830271209

[bib12] LanzavecchiaA1993 Identifying strategies for immune intervention Science 260 937944 8493532 10.1126/science.8493532

[bib13] LudewigBOchsenbeinAFOdermattBPaulinDHengartnerHZinkernagelRM2000 Immunotherapy with dendritic cells directed against tumor antigens shared with normal host cells results in severe autoimmune disease J Exp Med 191 795804 10704461 10.1084/jem.191.5.795 PMC2195849

[bib14] MärtenAZiskeCSchöttgerBWeineckSButtgereitPSchakowskiFvon RückerAScheffoldCSauerbruchTSchmidt-WolfI2001 Transduction of dendritic cells (DC) with CIITA gene:Increase of immunostimulatory activity of DC Cancer Gene Ther 8 211218 11332992 10.1038/sj.cgt.7700292

[bib15] MackensenADragerRSchlesierMMertelsmannRLindemannA2000 Presence of IgE antibodies to bovine serum albumin in a patient developing anaphylaxis after vaccination with human peptide-pulsed dendritic cells Cancer Immunol Immunother 49 152156 10881694 10.1007/s002620050614 PMC11036962

[bib16] MorseMAColemanREAkabaniGNiehausNColemanDLyerlyHK1999 Migration of human dendritic cells after injection in patients with metastatic malignancies Cancer Res 59 5658 9892184

[bib17] MortariniRAnichiniADi NicolaMSienaSBregniMBelliFMollaAGianniAMParmianiG1997 Autologous dendritic cells derived from CD34+ progenitors and from monocytes are not functionally equivalent antigen-presenting cells in the induction of melan-A/Mart-1(27-35)-specific CTLs from peripheral blood lymphocytes of melanoma patients with low frequency of CTL precursors Cancer Res 57 55345541 9407964

[bib18] MuldersPPangSDannullJKabooRHinkelAMichelKTsoCLRothMBelldegrunA1998 Highly efficient and consistent gene transfer into dendritic cells utilizing a combination of ultraviolet-irradiated adenovirus and poly(L-lysine) conjugates Cancer Res 58 956961 9500456

[bib19] MurphyGTjoaBRagdeHKennyGBoyntonA1996 Phase I clinical trial: T-cell therapy for prostate cancer using autologous dendritic cells pulsed with HLA-A0201-specific peptides from prostate-specific membrane antigen Prostate 29 371380 8977634 10.1002/(SICI)1097-0045(199612)29:6<371::AID-PROS5>3.0.CO;2-B

[bib20] NestleFOAlijagicSGillietMSunYGrabbeSDummerRBurgGSchadendorfD1998 Vaccination of melanoma patients with peptide- or tumor lysate-pulsed dendritic cells Nat Med 4 328332 9500607 10.1038/nm0398-328

[bib21] RissoanMCSoumelisVKadowakiNGrouardGBriereFde WaalMLiuYJ1999 Reciprocal control of T helper cell and dendritic cell differentiation Science 283 11831186 10024247 10.1126/science.283.5405.1183

[bib22] RomaniNGrunerSBrangDKampgenELenzATrockenbacherBKonwalinkaGFritschPOSteinmanRMSchulerG1994 Proliferating dendritic cell progenitors in human blood J Exp Med 180 8393 8006603 10.1084/jem.180.1.83 PMC2191538

[bib23] RosenbergSAYangJCSchwartzentruberDJHwuPMarincolaFMTopalianSLRestifoNPDudleyMESchwarzSLSpiessPJWunderlichJRParkhurstMRKawakamiYSeippCAEinhornJHWhiteDE1998 Immunologic and therapeutic evaluation of a synthetic peptide vaccine for the treatment of patients with metastatic melanoma Nat Med 4 321327 9500606 10.1038/nm0398-321 PMC2064864

[bib24] SallustoFLanzavecchiaA1994 Efficient presentation of soluble antigen by cultured human dendritic cells is maintained by granulocyte/macrophage colony-stimulating factor plus interleukin 4 and downregulated by tumor necrosis factor alpha J Exp Med 179 11091118 8145033 10.1084/jem.179.4.1109 PMC2191432

[bib25] Schmidt-WolfGDSchmidt-WolfIG1996 Cancer and gene therapy Ann Hematol 73 207218 8959938 10.1007/s002770050231

[bib26] Schmidt-WolfIGHuhnDNeubauerAWittigB1994 Interleukin-7 gene transfer in patients with metastatic colon carcinoma, renal cell carcinoma, melanoma, or with lymphoma Hum Gene Ther 5 11611168 7530496 10.1089/hum.1994.5.9-1161

[bib27] SchottMSeisslerJLettmannMFouxonVScherbaumWAFeldkampJ2001 Immunotherapy for medullary thyroid carcinoma by dendritic cell vaccination J Clin Endocrinol Metab 86 49654969 11600571 10.1210/jcem.86.10.7949

[bib28] SchulerGSteinmanRM1997 Dendritic cells as adjuvants for immune-mediated resistance to tumors J Exp Med 186 11831187 9379142 10.1084/jem.186.8.1183 PMC2199101

[bib29] SchwartzRH1990 A cell culture model for T lymphocyte clonal anergy Science 248 13491356 2113314 10.1126/science.2113314

[bib30] SetteAVitielloARehermanBFowlerPNayersinaRKastWMMeliefCJOseroffCYuanLRuppertJ1994 The relationship between class I binding affinity and immunogenicity of potential cytotoxic T cell epitopes J Immunol 153 55865592 7527444

[bib31] SteinbrinkKJonuleitHMullerGSchulerGKnopJEnkAH1999 Interleukin-10-treated human dendritic cells induce a melanoma-antigen-specific anergy in CD8(+) T cells resulting in a failure to lyse tumor cells Blood 93 16341642 10029592

[bib32] SteinmanRM1991 The dendritic cell system and its role in immunogenicity Annu Rev Immunol 9 271296 1910679 10.1146/annurev.iy.09.040191.001415

[bib33] ThomasRChambersMBoytarRBarkerKCavanaghLLMacFadyenSSmithersMJenkinsMAndersenJ1999 Immature human monocyte-derived dendritic cells migrate rapidly to draining lymph nodes after intradermal injection for melanoma immunotherapy Melanoma Res 9 474481 10596914 10.1097/00008390-199910000-00007

[bib34] ThurnerBHaendleIRoderCDieckmannDKeikavoussiPJonuleitHBenderAMaczekCSchreinerDvon den DrieschPBrockerEBSteinmanRMEnkAKampgenESchulerG1999a Vaccination with mage-3A1 peptide-pulsed mature, monocyte-derived dendritic cells expands specific cytotoxic T cells and induces regression of some metastases in advanced stage IV melanoma J Exp Med 190 16691678 10587357 10.1084/jem.190.11.1669 PMC2195739

[bib35] ThurnerBRoderCDieckmannDHeuerMKruseMGlaserAKeikavoussiPKampgenEBenderASchulerG1999b Generation of large numbers of fully mature and stable dendritic cells from leukapheresis products for clinical application J Immunol Methods 223 115 10037230 10.1016/s0022-1759(98)00208-7

[bib36] TimmermannJMCzerwinskiDKDavisTAHsuFJBenikeCHaoZMTaidiBRajapaskaRCasparCBOkadaCYvan BeckhovenALilesTMEnglemanEGLevyR2002 Idiotype-pulsed dendritic cell vaccination for B-cell lymphoma: clinical and immune response in 35 patients Blood 99 15171526 11861263 10.1182/blood.v99.5.1517

[bib37] TütingTWilsonCCMartinDMKasamonYLRowlesJMaDISlingluffCLWagnerSNvan der BruggenPBaarJLotzeMTStorkusWJ1998 Autologous human monocyte-derived dendritic cells genetically modified to express melanoma antigens elicit primary cytotoxic T cell responses in vitro: enhancement by cotransfection of genes encoding the Th1-biasing cytokines IL-12 and IFN-alpha J Immunol 160 11391147 9570527

[bib38] WestermannJAicherAQinZCayeuxZDaemenKBlankensteinTDorkenBPezzuttoA1998 Retroviral interleukin-7 gene transfer into human dendritic cells enhances T cell activation Gene Ther 5 264271 9578847 10.1038/sj.gt.3300568

